# An Assessment of Mesoporous Silica Nanoparticle Architectures as Antigen Carriers

**DOI:** 10.3390/pharmaceutics12030294

**Published:** 2020-03-24

**Authors:** Xinyue Huang, Helen E Townley

**Affiliations:** 1 Nuffield Department of Women’s and Reproductive Health, Oxford University, John Radcliffe Hospital, Oxford OX3 9DU, UK; chch41hxy@gmail.com; 2 Department of Engineering Science, Oxford University, Parks Road, Oxford OX1 3PJ, UK

**Keywords:** nanoparticle, silica, adjuvant, mesoporous, vaccine

## Abstract

Mesoporous silica nanoparticles (MSNPs) have the potential to be used as antigen carriers due to their high surface areas and highly ordered pore network. We investigated the adsorption and desorption of diphtheria toxoid as a proof-of-concept. Two series of nanoparticles were prepared—(i) small pores (SP) (<10 nm) and (ii) large pores (LP) (>10 nm). SBA-15 was included as a comparison since this is commercially available and has been used in a large number of studies. External diameters of the particles ranged from 138 to 1509 nm, surface area from 632 to 1110 m^2^/g and pore size from 2.59 to 16.48 nm. Antigen loading was assessed at a number of different ratios of silica-to-antigen and at 4 °C, 20 °C and 37 °C. Our data showed that protein adsorption by the SP series was in general consistently lower than that shown by the large pore series. Unloading was then examined at 4 °C, 20 °C and 37 °C and a pH 1.2, 4.5, 6.8 and 7.4. There was a trend amongst the LP particles towards the smallest pores showing the lowest release of antigen. The stability of the MSNP: antigen complex was tested at two different storage temperatures, and storage in solution or after lyophilization. After 6 months there was negligible release from any of the particles under any of the storage conditions. The particles were also shown not to cause hemolysis.

## 1. Introduction

Nanosystems could play a role in vaccine technology by enabling targeted delivery and controllable release for specific cellular immunity. They could also protect antigens from enzymatic degradation and denaturation thereby allowing a prolonged antigen release for long-lasting humoral response. It has also been suggested that the size and structure of nanoparticles could elicit an immune response due to their size and shape being similar to those of bacteria and viruses [1,2,3].

To improve the efficacy of preventative vaccines one challenge is to improve adjuvants to overcome the current limitations. It has been reported that mesoporous silica nanoparticles (MSNPs) possess intrinsic immunogenicity [4]. Furthermore, MSNPs have been considered an ideal candidate due to their stability, the protection of the antigen, potentially high binding capacity, high biocompatibility, tunable architecture and low cost [5,6]. This work therefore focuses on the capacity of different architectures of silica nanoparticles to carry antigen. The adsorption of large antigens such as proteins and peptides will be particularly affected by the architecture of the silica. A high protein loading capacity would reduce the amount of silica nanoparticles needed in vivo, while still maintaining a high antigen dose.

Many vaccines are live, attenuated variants of a pathogen. This results in long-lived immunity similar to natural infection but with mild, usually asymptomatic infection. For many pathogens, for example influenza, this is not possible since the natural infection does not confer adequate immunity [7]. For such vaccinations, non-living antigens are used. However, these are poorly immunogenic and require additional stimulation to generate immunity. Therefore, adjuvants are used to increase the magnitude of the response to the vaccine by increasing antibody titer [8]. Adjuvants may also be used to increase seroconversion rates in populations such as the elderly or infants, due their reduced responsiveness [9,10]. Furthermore, adjuvants can enable the use of smaller doses of antigen since comparable responses can be obtained; which could be important in terms of either limited amounts of antigen or cost. The adjuvant may also alleviate the need for repeated immunizations, which can improve patient compliance, due to a reduction in the number of doses needed for protection [11,12,13].

While adjuvants can significantly increase the immune response and are often included in vaccination protocols, only a few types of adjuvants are currently used in vaccines approved for human use. These include metal salts (aluminum and calcium salts), oil emulsions (Freunds complete or incomplete and MF59) and bacterial derivatives [1,14,15]. However, the commonly used alum has semi-particulate hydrogel properties which means that it cannot be frozen or lyophilized, thereby limiting shelf life [16]. Given the limited number of options and the concerns over aluminum adjuvants [17], other agents which can increase the immune response are actively sought. Previous studies have demonstrated that the well-known MSNP SBA-15 can act as an adjuvant [18]. Therefore, MSNPs could present an ideal substrate on which to load antigen and to have intrinsic adjuvant activity.

A number of different engineered nanomaterials have been studied for their potential to enhance the delivery of antigens to the immune system. These include materials such as gold, silver, and chitosan (see Reference [19] for full details). It is likely that metals will have superior adjuvant potential but persistence in the body may be of concern. Polymeric nanoparticles are easy to synthesize, biocompatible and biodegradable. Liposomes may also be considered and while the liposome itself may have low immunostimulatory effects, it could enhance cellular uptake of antigens. In addition to varying the material of the nanoparticle, the surface chemistry can also be manipulated. Antigen uptake could be varied by changing the surface charge, hydrophobicity and functional groups for antigen presenting cell (APC) targeting [20]. Due to the negatively charged hydrophilic outer surface of cell membranes, it is likely that positively charged particles might have higher binding affinity than neutral or negatively charged nanoparticles [21,22].

To investigate the influence of the silica architecture on the loading, unloading, and long-term stability of an antigen we made a series of particles with different external diameters and pore sizes. Loading and unloading were performed at a series of different temperatures, pH and silica-to-antigen ratios. The stability of the antigen after adsorption to the particles was followed over 6 months under a range of different storage conditions.

## 2. Experimental

A number of different mesoporous silica nanoparticles (MSNPs) with varying external diameter and porosity were synthesized as follows. One set of nanoparticles had small pores (SP) and the other much larger pores (LP). Santa Barbara Amorphous particles (SBA-15) were synthesized in-house by Cristalia Produtos Quimicos (Sao Paulo, Brazil). SBA-15 was used for comparison purposes since it has often been used in the literature.

### 2.1. Synthesis Protocol of MSNPs

The synthesis protocol for all MSNPs is described in full detail in our previously published work [23]. After synthesis, the template is removed from the particles by resuspension in acidified methanol (40 mL methanol; 2 mL 37% hydrochloric acid). The sample was then refluxed at 80 °C for 24 h. Subsequently, the suspension was cooled to room temperature and particles collected by centrifugation and washed with ethanol. The particles were dried for at least 24 h in a desiccator under high vacuum at room temperature. The particles were then ground to a fine powder using a mortar and pestle.

### 2.2. Scanning Electron Microscopy (SEM)

Secondary scatter electron images of the specimens were taken using a Carl Zeiss Evo LS15 VP-Scanning Electron Microscope (Zeiss, Jena, Germany). The specimens were prepared as either dry-cast or dry-spray. For dry-cast samples, the particles were suspended in ethanol and sonicated for 2 min. Sample (50 µL) was dropped onto separated carbon adhesive discs (Agar Scientific, Standsted, UK) on short pin specimen stubs. After evaporation of the solvent the sample was dry-cast on the sticker. For the dry-spray samples, approximately 1 mg of particles was sprayed onto a carbon adhesive disc on a short pin specimen stub. Excess particles were cleaned using an air-duster. All specimens were then sputter-coated with 3 nm Pd–Au (under high vacuum in Argon, coat for 60 s at 20 mA) on a Quorum^®^ SC7620 sputter coater before the testing. Images were taken using SmartSEM interface (Zeiss^®^, Jena, Germany).

### 2.3. Transmission Electron Microscopy (TEM)

Bright field transmission electron microscope (TEM) images of the specimens were taken using a JEOL JEM-3000F FEGTEM. Samples were suspended at 125 µg/mL in ethanol and dry-cast onto holey carbon coated copper TEM grid (Agar Scientific, Stansted, UK). Images were taken using DigitalMicrograph™ (Gatan Inc., Pleasanton, CA, USA). A single tilt TEM specimen holder was used and a 4k Gatan Ultrascan camera (Gatan Inc., Pleasanton, CA, USA).

### 2.4. Surface Physical Properties Measurement

The specific surface area of MSNPs was assessed by nitrogen adsorption–desorption isotherm measurements on a Gemini VI or TriStarII Plus (Micromeritics, Norcross, GA, USA) surface analyzer at −196 °C and calculated with Brunauer-Emmett-Teller (BET) theory [24]. Specimens were degassed with nitrogen at 50 °C overnight before testing. BET surface area was calculated from isotherm adsorption data at P/P_0_ from 0.05 to 0.30 (linear region):

(1) $${\mathrm{SA}}_{\mathrm{BET}}=\frac{CSA\times N_{A}}{22414\times 10^{18}\times \left(S+Y_{INT}\right)\;}\;,$$

where SA_BET_ is BET surface area (m^2^/g); CSA is the analysis gas molecular cross-sectional area (0.162 nm^2^ for N_2_); N_A_ is Avogadro constant 6.023 × 10^23^; S is the slope (g/cm^3^); Y_INT_ is the Y-intercept (g/cm^3^).

Porosity (pore volume and pore size distribution) was evaluated by using the Barrett, Joyner and Halenda (BJH) method from both the absorption and desorption branch [25].

### 2.5. Disc Centrifuge Measurement

The hydrodynamic particle size distributions were determined using a Disc Centrifuge (DC24000; CPs instrument). Prior to measurements, a sucrose gradient was built and polyvinyl chloride (PVC) particle calibration standards were applied (266nm; PVC000266, Analytik Ltd., Cambridge, UK).

### 2.6. Surface Mobility Measurement

The zeta (ζ) potential of the MSNPs was measured using a Zetasizer Nano ZS (Malvern, UK). To determine the ζ potential or electrokinetic potential, the MSNPs were suspended in ddH_2_O (pH 7.0) prior to the measurement. Samples were placed into DTS1070 disposable capillary cells for measurement. After 120 s equilibrium time, 30 runs were read before the calculation of electrophoretic mobility, zeta potential and zeta potential distribution. Nanoparticles having a Zeta potential between 15 mV and 30 mV (or equivalent negative values) are considered moderately stable and those with values above 30 mV (or equivalent negative values) to have good stability.

### 2.7. Antigen Loading onto MSNPs

Diphtheria toxoid (Fundação Butantan, Sao Paulo, Brazil) was loaded onto the different test samples of MSNPs. The stock concentration of diphtheria toxoid was 10 mg/mL. Before loading, the MSNP samples were sonicated in phosphate buffered saline (PBS; pH 7.4). The stock concentration of MSNPs was 50 mg/mL. To gain different loading ratios the particles were loaded with diphtheria toxoid according to Table 1.

All samples were produced in triplicate. The mixtures were vortexed for 30 s followed by incubation for 40 h at room temperature on a rocking table. A negative control comprising only diphtheria toxoid at a concentration of 250 µg/mL with no MSNP addition was prepared at the same time.

#### 2.7.1. Loading Temperature Variation

The loading experiment was also repeated by incubating the samples under the same conditions described in Section 2.7, at 4 °C and 37 °C.

#### 2.7.2. Assessment of Loading Efficiency

After the loading of the MSNPs with diphtheria toxoid, the nanoparticles were collected by centrifugation at 10,000 rpm for 1 min. The amount of unbound diphtheria toxoid in the supernatant was determined from a Bradford’s assay (Section 2.10) and was used to calculate the loading efficiency.

### 2.8. Assessment of Release of the Diphtheria Toxoid from the MSNPs

Release of the diphtheria toxoid was assessed for the different MSNPs which had been loaded at a ratio of 1:20 and also the control particle SBA-15 which had been loaded at a ratio of 1:40. The loading had been achieved at 4 °C for 24 h in PBS.

Release of the diphtheria toxoid was assessed at pH 4.5, 6.8, 7.4 and at both room temperature, 4 °C and 37 °C. Loaded MSNPs were added to 450 μL of buffer in triplicate for each test condition. Samples (10 μL) were collected at times 0, 15 min, 30 min, 45 min, 60 min, 2 h, 3 h, 6 h, 12 h, 24 h, 48 h and 72 h. Fresh PBS (10 μL) was added after each removal. Samples were centrifuged and the supernatant collected. The amount of protein in the supernatant was determined from a Bradford’s assay (Section 2.10).

### 2.9. Stability Test

To test the stability of the different MSNPs adsorbed with the diphtheria toxoid, the complexes were kept under four different storage conditions: (i) in suspension at 4 °C, (ii) in suspension at room temperature, (iii) lyophilized at 4 °C, and (iv) lyophilized at room temperature.

MSNPs were resuspended to a final concentration of 40 mg/mL for LP2 and LP3, and 80 mg/mL for SBA-15, in PBS. Therefore, the diphtheria toxoid: MSNP ratio would be 1:20 for LP2 and LP3, and 1:40 for SBA-15. The samples were incubated at 4 °C to allow adsorption to occur. Samples were then aliquoted (125 μL lots). Half the samples were then kept in suspension at either room temperature or 4 °C. The other half were lyophilized in a freeze drier (Mini Lyotrap, LTE Scientific Ltd., Oldham, UK). The lyophilized samples were then either kept at room temperature or 4 °C.

At time points between 0 and 6 months the samples were analyzed. For conditions (i) and (ii) to each tube containing 125 μL, a further 125 μL of PBS was added and the sample resuspended. For conditions (iii) and (iv) 250 μL of PBS was added to each tube and the sample resuspended. All samples were then centrifuged at 10,000 rpm for 1 min and the supernatant collected (i.e., released antigen). To release the remaining diphtheria toxoid from the nanoparticle, the nanoparticles were subsequently resuspended in 2% SDS and boiled at 95 °C for 5 min. The samples were then centrifuged at 10,000 rpm for 1 min and the supernatant collected (i.e., liberated antigen).

### 2.10. Protein Concentration Determination

Protein samples were quantified using Bradford’s reagent (Bio-Rad Laboratories, Watford, UK). Dye reagent is prepared by diluting 1 part dye reagent concentrate with 4 parts deionized water. Diluted dye reagent (5mL) was added to a clean, dry tube. Either standard or solution (100 μL) was added to the tube. Samples were prepared in triplicate. The tubes were vortexed and incubated at room temperature for 5 min. Absorbance of the dye was quantified in a plate reader (Tecan Infinite 200) at 590 nm. A standard curve (R^2^ value > 0.98) was generated using diluted Bovine Serum Albumin protein standards from a 2 mg/mL stock.

Samples which contained SDS were assessed using the BCA protein assay kit (Pierce, Thermo Scientific, Gloucester, UK) as per the manufacturer’s instructions

### 2.11. SDS-PAGE

SDS-polyacrylamide gels from Thermo Scientific (Novex 8–16% Tris-glycine mini gels) were assembled in a Bio-Rad Mini Protean II system and 1× Novex Tris-Glycine SDS running buffer was added to the top and bottom reservoirs. Immediately prior to loading, the samples were mixed with an equal volume of Novex Tris-Glycine SDS sample buffer and incubated at 98 °C for 5 min. Ten micrograms of each sample was loaded into the wells of the gel and 10 μL of Page Ruler pre-stained protein ladder (10 to 180 kDa) was loaded into the first well. The gel was run at 100 V until the dye front had reached the bottom of the gel. To visualize the proteins, the gel was stained with Coomassie Brilliant Blue solution (0.5 g Coomassie brilliant blue R250 was dissolved in 200 mL methanol and 160 mL distilled water, followed by the addition of 40 mL acetic acid). The gel was soaked in staining solution for approximately 30 min. The gel was de-stained in 20% (*v*/*v*) methanol and 10% (*v*/*v*) acetic acid, overnight.

### 2.12. Haemotoxicity Assay

Defibrinated horse blood was used to assess the hemotoxicity of the MSNPs. 10 mL blood was centrifuged at 2000 g for 5 min. The pellet of blood cells was washed three times using PBS. After the final wash the supernatant was removed and the pellet resuspended in 1:10 (*v*/*v*) PBS. The sample was then divided into equal volumes in separate tubes. As a positive control 400 µL water was added to the sample and a negative control used the same volume of PBS. Different MSNPs were added at 0.05 mg/mL in 400 µL PBS; the samples were fully resuspended using a probe sonicator for 2 min at 80% full amplitude (Sonics vibracell; 130 W, Sonics & Materials Inc., Newtown, CT, USA). All samples were then incubated with blood at 37 °C for an hour. After incubation, the samples were centrifuged at 10,000g for 5 min. Supernatant (100 µL) was taken from each tube and placed in a 96 well plate. The absorbance was measured at 595 nm. The experiment was repeated on three separate occasions.

## 3. Results

### 3.1. Physical Characterization of the MSNPs

MSNPs have the potential to be used as antigen carriers due to the highly ordered pore network which is homogeneous and allows fine control of antigen adsorption, and the high surface areas which permit greater antigen adsorption [26]. Two series of nanoparticles were prepared; those with small pores (<10 nm) and large pores (>10 nm). SBA-15 was also included as a comparison since this is commercially available and has been used in a large number of studies in the literature.

**Figure 1:**
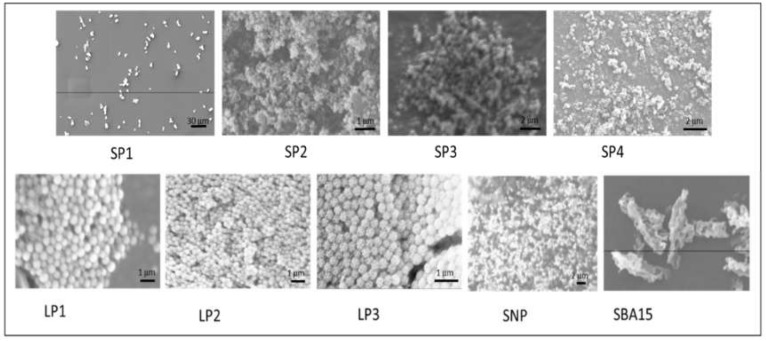
Characterization of synthesized silica nanoparticles using scanning electron microscopy. Solid nanoparticles (SNP) and SBA-15 are shown for comparison. Scale bars shown are (SP1) 30 μm, (SP2) 1 μm, (SP3, SP4) 2 μm, (LP1, LP2, LP3) 1 μm, (SNP) 2 μm.

**Figure 2:**
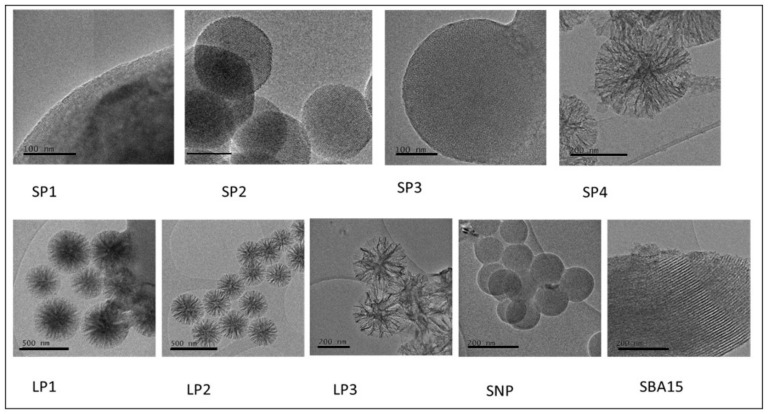
Characterization of synthesized silica nanoparticles using transmission electron microscopy. Solid nanoparticles (SNP) and the SBA-15 are shown for comparison. Scale bars (SP1-SP3) 100 nm; (SP4) 200 nm; (LP1) 500 nm; (SNP) 200 nm; (SBA-15) 200 nm.

The external diameter of the nanoparticles was measured by disc centrifuge and the SP series ranged from 138 nm to 1509 nm, whereas the LP series ranged from 217 nm to 462 nm (Table 2). The zeta potential of the nanoparticles showed all the particles to be negatively charged which would be expected due to the hydroxyl groups on the surface of the silica. The particles all had a large surface ranging from 162 to 1110 m^2^/g. Those particles in the SP group had pores ranging from 2.59 nm to 2.94 nm and the LP group had pores ranging from 11.15 nm to 16.48 nm. The pore volume was greater for those particles with larger pore sizes (except for SP4). It should be noted that two peaks were visualized in the BJH pore peak size plot for LP1, LP2 and LP3 for example, 4 nm and 70 nm for LP1.To assess the morphology of the particles, scanning electron microscopy (SEM) and TEM images were collected (Figure 1 and Figure 2). SP1, SP2 and SP3 showed spherical morphology with hexagonally ordered packing of pores seen in the first two and less ordered packing seen in SP3. The mesostructure of LP1, LP2 and LP3 was very different from the SP series of particles. Here, the discrete pores are replaced with a more open, lacy structure without ordered pores. The solid nanoparticles (SNP) have no pores and are used as a control. SBA15 is a mesoporous silica sieve based on uniform hexagonal pores with a narrow pore size distribution.

### 3.2. Antigen Loading

**Figure 3:**
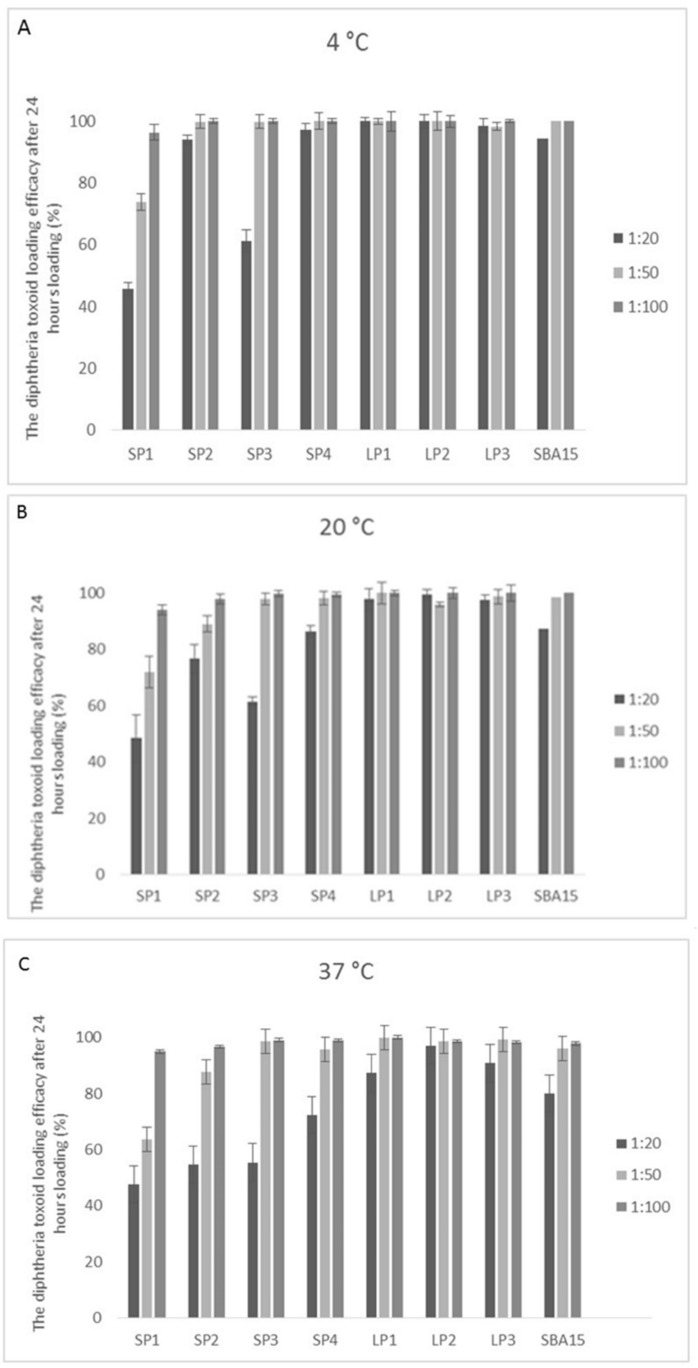
Antigen loading onto the MSNPs. Diphtheria toxoid was incubated with the different MSNPs for 24 hours at either (**A**) 4 °C; (**B**) 20°C; (**C**) 37 °C. The toxoid to MSNP ratio was varied from 1:20, 1:50, and 1:100. Data shown is mean ± standard deviation. Three independent experiments were performed with triplicate samples, and data from all experiments combined.

The encapsulation of antigenic proteins into a silica nanoparticle carrier system mainly takes place through adsorption [5]. In order to determine the level of protein adsorption, after the incubation period of the particles with the antigen, the antigen-adsorbed samples are centrifuged and the unbound protein remaining in the supernatant is measured and compared to the pre-adsorption concentration using a colorimetric protein assay. It can be seen from Figure 3 that in general, the lower temperature (4 °C) resulted in more efficient adsorption than higher temperatures (20 °C, 37 °C). Also, the general trend showed that the lower the ratio of particle: antigen the greater the adsorption. This is most likely due to the availability of an increased total amount of antigen available for binding.

The lowest adsorption was seen with SP1 at a loading ratio of 1:20 at 37 °C (45.7 ± 9.2%), although SP2 and SP3 are statistically not significantly better (*p* > 0.05) under the same conditions. However, it can be seen that loading efficiency can be drastically improved by changing the loading conditions: an efficiency of 96.4 ± 2.8% can be achieved at 4 °C when the loading ratio is changed to 1:100. The effect of the temperature during loading was minimal for SP1 and SP3. A similar trend was seen in all of the small pore series, although it was less pronounced in SP2 and SP4.

The large pore series showed consistently higher loading efficiency, although the lowest adsorption was once again seen at 37 °C. It is likely that the larger pores allow more cargo to be carried on the surface of the particles, hence more of the protein is removed from the loading solution.

The SBA-15 showed intermediate loading potential between the small pore and large pore series of particles (the lowest binding seen was 80.2 ± 4.1% for SBA-15, compared to the lowest binding seen for LP1 of 87.3 ± 1.9%, and 45.7 ± 9.2% for SP1).

We also assessed the degree of binding of the toxoid to non-porous solid silica nanoparticles (SNPs) of different sizes (Figure S3). The SNPs ranged in size from 275 nm to 847 nm. Binding was very low as expected, and the highest binding of approximately 20% was shown for the 588 nm particles with 1:100 loading at 37 °C.

It is important to optimize the process since a low loading efficiency can be wasteful, which is especially important if the cargo is expensive or difficult to produce. The higher loading efficiency at lower loading ratios may simply indicate that the particle has reached loading capacity and the excess remains in the media.

### 3.3. Antigen Unloading from MSNPs

Since the large pore series appear to load in a more consistent manner, irrespective of the loading ratio and loading temperature, unloading experiments were only performed for this series of particles. Unloading of the particles was examined at 4 °C (cold storage), 20 °C (room temperature) and 37 °C (body temperature) and at pH 1.2, 4.5, 6.8 and 7.4. The acidity of the solutions was chosen to represent the empty stomach (pH 1.2), full stomach (pH 4.5) saliva (pH 6.8) and blood (pH 7.4). A number of different time points were examined but the peak of release was seen at around 3 h (see Figure S2a–d).

**Figure 4:**
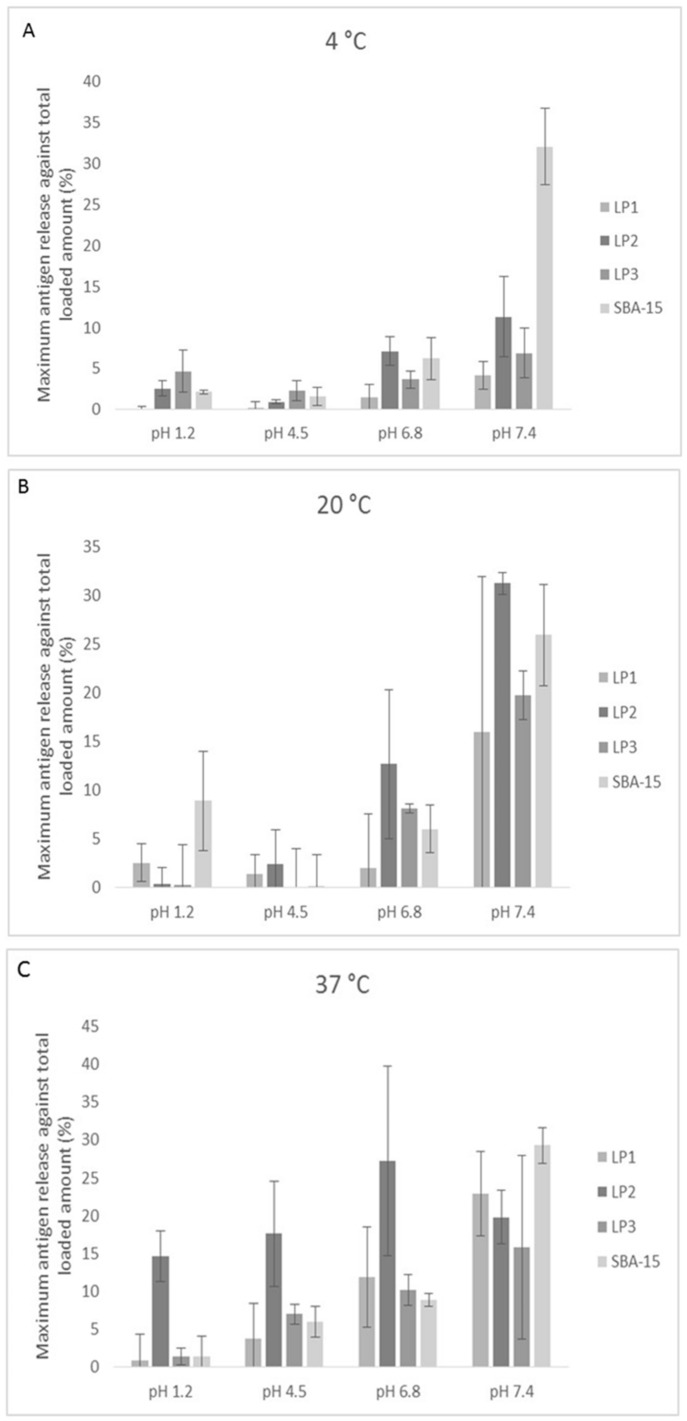
Antigen unloading from the MSNPs. Diphtheria toxoid loaded MSNPs were incubated in PBS buffer for 3 hours at either (**A**) 4 °C, (**B**) 20 °C, or (**C**) 37 °C. The release buffer was varied from pH 1.2, pH 4.5, and pH 7.4. Data shown is mean ± standard deviation. Three independent experiments were performed with triplicate samples, and data from all experiments combined.

As a general trend it can be seen that release of the antigen from all of the three large pore particles and SBA-15 is greater with increasing pH (Figure 4); for example, LP2 ranges from a minimum release of 0.35 ± 1.68% (pH 1.2; 20 °C) to a maximum release of 31.2 ± 1.2% (pH 7.4; 20 °C); an 89 fold increase (*p* < 0.005). The isoelectric point of the diphtheria toxoid is 4.1 [27], therefore the diphtheria toxoid would be positively charged in lower pH conditions. Since silica is negatively charged, a more positively charged molecule would be more likely to bind to the nanoparticle and therefore less likely to be released. The release of the toxoid from the particle appears to be more influenced by the pH than by temperature. Unloading of the particles is in general greatest at pH 6.8 and 7.4, indicating that release would be higher in the mouth and blood, rather than the stomach. At body temperature LP2 also shows substantial release at pH 1.2 and 4.5.

### 3.4. Stability of the MSNP: Antigen Complex

**Figure 5:**
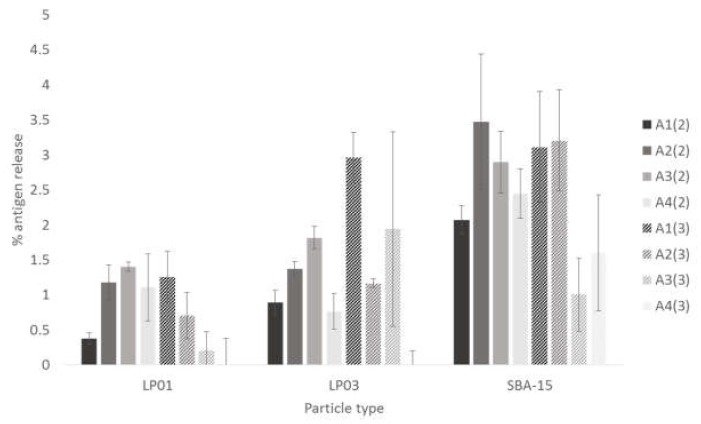
Determination of toxoid leached from silica nanoparticles. The supernatant was assayed to determine the protein leached under various different storage conditions. The samples were kept under the following conditions (A1) in suspension, kept at 4 °C; (A2) in suspension, kept at RT; (A3) lyophilized, kept at 4 °C; (A4) lyophilized, kept at RT. Numbers in brackets indicate incubation for either (2) two months, or (3) three months. Data shown is mean ± standard deviation.

To test the stability of the MSNP: antigen complex, the construct was tested under two different storage temperatures. It was also tested in solution and lyophilized. The amount of antigen released into solution during storage (or after resuspension in the latter case) was assessed using a protein assay. At *t* = 0, 5.4% of the loaded antigen was released from SBA-15 (lyophilized, 4 °C) but all other complexes and storage conditions showed no release (data not shown). At *t* = 1 month, once again the only detectable release was from SBA-15 after lyophilization and storage at 4 °C (data not shown). After 2- and 3-months storage there was very low release of the antigen from the silica. LP01 showed a maximum release of less than 1.5% for all storage conditions (Figure 5). LP03 showed a maximum release of 3% for all storage conditions, whereas SBA-15 showed the highest release albeit still under 3.5% (Figure 5).

**Figure 6:**
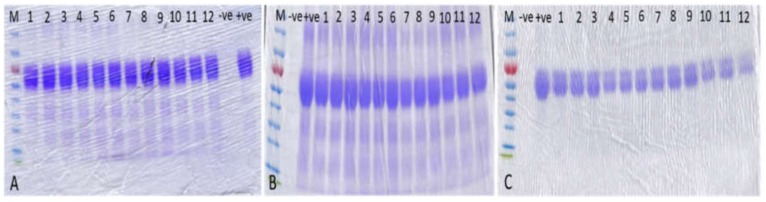
Stability test on released toxoid. SDS-PAGE analysis of protein released from the various MSNPs after (**A**) 0 months (**B**) 3 months (**C**) 6 months. The samples were kept under the following conditions A1: In suspension, kept at 4 °C, A2: In suspension, kept at RT, A3: Lyophilised, kept at 4 °C, A4: Lyophilised, kept at RT. Lanes are loaded as follows: (M) Marker, (−ve) Negative control, (+ve) Positive control, (1) LP2@A1, (2) LP3@A1, (3) SBA-15@A1, (4) LP2@A2, (5) LP3@A2, (6) SBA-15@A2, (7) LP2@A3, (8) LP3@A3, (9) SBA-15@A3, (10) LP2@A4, (11) LP3@A4, (12) SBA-15@A4.

To analyze the quality of the protein that was bound to the MSNPs, at each time point the toxoid was liberated from the particles using surfactant and heat treatment. The protein was then loaded onto SDS-PAGE to look for breakdown of the protein. At *t* = 0, 1,2, 3, 6 months there was no apparent difference between the native antigen and that which had been bound to the particles, under any of the storage conditions (Figure 6). This indicates that the silica adsorbed toxoid will remain bound and relatively unchanged under a variety of storage conditions (Figure 5 and Figure 6).

To test for hemocompatibility, the different silica nanoparticles were tested for their interactions with red blood cells (Figure S1). The particles were tested with and without adsorbed diphtheria toxin. Water was included as a positive control, which would cause cell lysis. PBS was included as a negative control. It can be clearly seen that none of the silica nanoparticles, with or without diphtheria toxin, caused any degree of hemolysis.

## 4. Discussion

Our data shows that the architecture of the nanoparticles affects the adsorption of the antigen and that the loading and unloading conditions greatly affect the interaction. The potential of MSNPs as vaccine adjuvants was first observed by Mercuri et al. [18] in a study using SBA-15 as a carrier and adjuvant for Int1β, a bacterial recombinant protein.

Mesoporous silica nanoparticles are excellent candidates for antigen adsorption due to their high surface area. Those in our study ranged from 162 m^2^/g to 1110 m^2^/g. The most common form of MSNPs studied have hexagonal symmetry; and cargo can be easily loaded into the cylindrical pores [4]. The SBA-15 used in this study was synthesized in-house. The material has been cited in the literature as having hexagonal pore morphology and aggregated particles with size varying from 800 nm to 2 μm, with a pore size of approximately 8 nm, 0.8–1.00 cm^3^/g pore volume and a surface area of 450–550 m^2^/g [28]. Our study showed very similar parameters with an average pore size of 7.7–7.9 nm and a pore volume of 1.3 cm^3^/g. The thickness of the walls has been cited as 3.1–6.4 nm, which gives the material a higher hydrothermal and mechanical stability than, for example, MCM-41 [26]. It has been suggested that such large particles may have limitations in large molecular diffusion and adsorption capacity [29]. SBA-15 is much larger than the other particles investigated here and is also a rod-shaped particle rather than spherical. The SBA-15 is designated as belonging to the SP group in our study. The LP group showed more consistently high loading across the different temperatures and pH range tested. Previous experiments have shown with SBA-15 [18] that there was an increased immune response in mice, although there was no evidence to suggest that the protein was released from the carrier. Later work examined the uptake and release of protein by hollow MSNPs [30]. They showed that the adsorption of up to 150 μg/mg nanoparticles took place via a two-step pattern; rapid adsorbance of the protein was observed over the first two hours, followed by a second slower loading phase over the next 30 h. The release kinetics of the protein showed rapid release in the first 12 h, followed by release of up to 50% over the next 6 days. Protein adsorption has also been shown to be dependent upon the pore entrance and cavity size [31]. Our data clearly shows that protein adsorption by the SP series with pores less than 10 nm was, in general, consistently lower.

Although not statistically significant, there seemed to be a trend amongst the LP particles towards the smallest pores showing the lowest release of antigen. The adsorption and desorption of a protein from a silica particle is not simple and has recently been discussed by McUmber et al. [32]. These findings suggested that adsorption kinetics were dominated by energy barriers associated with electrostatic interactions but once adsorbed, protein–surface interactions were dominated by short-range non-electrostatic interactions. Adsorption of the protein herein was performed in PBS, pH7.4, however this may not have been optimal given that the IP of the protein is 4.1. Other studies using human PSA antigen [33] and two monoclonal IgGs [34] have shown absorbance maxima for binding to silica surfaces at around the isoelectric points of the antibody. Therefore the generally greater release of the antigen at pH 6.8 and 7.4 would not be unexpected. Another group has also reported that the pH dependence of adsorption is influenced by the pH dependent variation in the self-association of the protein when in solution with the silica nanoparticles [35]. Furthermore, the pH of the solution will affect both the dynamic adsorption and the equilibrated adsorbed amount. 

The particles in our study were loaded with diphtheria toxoid. The molecule comprises three abutting domains that are connected by interdomain linkers. The N-terminal C domain, middle T domain and C-terminal R domain consist of residues 1–193, 205–378 and 386–535, respectively. Schematically diphtheria toxoid is Y-shaped with the base formed by the T domain, one arm of the Y formed by the C domain and the other arm formed by the R domain. The Y is about 90 Å high, 50 Å across the top of the Y but only 30 Å thick [36]. The SP series of particles have pore sizes ranging from 2.59–5.71 nm, whereas the LP series range from 11.15–16.48 nm (Table 2). Therefore, it can be clearly seen that there is a much greater chance that the antigen would rest within the pores of the LP series more easily. This was confirmed by the data shown in Figure 3 in which the LP series shows greater binding of the antigen at all 3 loading temperatures.

The immune response will be dependent upon the structure of the silica and the consequent release profile. Reducing the release rate of proteins from 48% (SBA-15) to less than 8% (430 nm MSNPs) has been shown to enhance the antibody titer [6]. In our study, LP3 or LP1 show the slowest release rate at 37 °C at pH 1.2, 4.5 and 6.8. At pH 7.4 there is no statistical difference between the samples tested. Higher release rates would be expected at higher pH since the isoelectric point of the toxoid is 4.1 and therefore the protein would be negatively charged at higher pH and therefore less likely to bind to negatively charged silica.

The biocompatibility of MSNPs depends on the particle size, morphology, structure, surface properties and the dosage. At lower concentrations MSNPs have been found to be non-toxic in a variety of cell lines but at higher concentrations they have inhibitory effects on cells [37,38,39]. It is thought that the surface of the particles dictates the toxicity of nanoparticles. Furthermore, smaller nanoparticles which have relatively larger surface areas and abundant silanol groups are more likely to produce ROS, which can cause cell injury [40]. Nevertheless, our data shows that, irrespective of size, none of the nanoparticles tested in our study show hemotoxicity in vitro.

While vaccinations are generally administered by subcutaneous or intramuscular injection, oral administration is preferred for a number of reasons. We had hypothesized that the adsorption of the antigen to the silica nanoparticle could protect the molecule from degradation in the harsh conditions of the stomach. However, analysis of the antigen by SDS-PAGE (data not shown) showed that the protein was degraded after 3 h incubation in simulated stomach conditions of a gastric juice solution (method after Donhowe et al. [41]). Nevertheless, long term storage was tested over a series of months after the silica- antigen complex was either lyophilized or kept in physiological buffer and kept at 4 °C or room temperature. This showed that there was no breakdown of the antigen for at least 3 months.

We have subsequently investigated the take up rate of MSNPs by an immortalized macrophage-like cell line [23]. The optimal external diameter for take up into the cells was shown to be 217 nm (LP3). When this particle was loaded with diphtheria toxoid, there was an internalization rate of 53%. The MSNPs were also shown to be generally biocompatible since cell viability was not altered by the loading of particles, either with or without antigen.

## 5. Conclusions

We have shown that mesoporous silica nanoparticles present a possible alternative to currently used adjuvants and could be loaded with toxoid for vaccination. A wide variety of mesoporous silica nanoparticles can be fabricated and the pore sizes manipulated and controlled. Of the particles tested those with large pore sizes showed greater antigen binding. Nanoparticles with bound toxoid could then be lyophilized and stored for at least 3 months without breakdown of the antigen. While the long-term fate of any adjuvant material is always of concern, silica material is biocompatible and will also degrade naturally in the body over a period of several months [42].

## Figures and Tables

**Table 1:** Antigen loading onto mesoporous silica nanoparticles (MSNPs). The table shows the volume diphtheria toxin (DT) stock solution, MSNP-PBS suspension and PBS added in a single test tube in the DT loading experiments. The initial concentration of both nominal DT and MSNPs is shown.

DT:MSNP	1:2	1:5	1:10	1:20	1:50	1:100
Volume of DT stock solution (µL)	10	10	10	10	10	10
Volume of MSNPs suspension (µL)	4	10	20	40	100	200
Volume of PBS (µL)	386	380	370	350	290	190
Total volume (µL)	400	400	400	400	400	400
Nominal DT concentration (µg/mL)	250	250	250	250	250	250
MSNPs concentration (µg/mL)	500	1250	2500	5000	12500	25000

**Table 2:** Summary of nanoparticle properties. Size is shown as the average hydrodynamic diameter of the particles ± standard deviation. Zeta potential, surface area and calculated pore sizes and pore volumes are also shown. N/A = not attained.

Morphology	Average Yield (%)	Size (nm)	Surface Properties						
			ζ Potential (mV)	BET Surface are (m^2^/g)	Average Pore Size (nm)		Pore Peak Size (nm)		Pore Volume (cm^3^/g)
					Ads.	Des.	Ads.	Des.	
SP1	62.4	1509 ± 269	−26.8	992.4	2.6	2.7	2.6	2.7	0.78
SP2	80.6	138 ± 26	−20.7	1110.9	2.9	2.9	2.6	2.8	1.02
SP3	96.5	496 ± 13	−29.5	162.3	2.9	2.9	2.4	2.7	0.13
SP4	88.5	202 ± 19	−24.0	632.1	5.7	4.7	5.5	3.8	0.77
LP1	88.6	462 ± 25	−20.6	701.9	11.1	10.2	4 & 70	4 & 70	1.90
LP2	60.1	217 ± 22	−22.9	650.0	12.4	13.0	3.5 & 40	3.5 & 40	1.80
LP3	91.9	217 ± 5	−16.8	660.0	16.5	16.2	3.5 & 70	3.5 & 70	2.40
SBA-15	N/A	N/A	−19.7	794.0	7.9	7.7	10.0	10.0	1.30

## Supplementary Materials

The following are available online at https://www.mdpi.com/1999-4923/12/3/294/s1, Figure S1: Assessment of hemolysis caused by incubation of blood with particles, Figure S2: Time course to show toxoid release, Figure S3: Antigen loading on solid silica nanoparticles.
