# Supplementary material for: An Assessment of Mesoporous Silica Nanoparticle Architectures as Antigen Carriers
Source: Pharmaceutics. 2020 Mar 24;12(3):294. doi: 10.3390/pharmaceutics12030294 (PMC7151194; doi:10.3390/pharmaceutics12030294)
Supplement: Supplementary file 1 [file pharmaceutics-12-00294-s001.pdf]

pharmaceutics

MDPI

# Supplementary Materials: An assessment of Mesoporous Silica Nanoparticle Architectures as Antigen Carriers

**Xinyue Huang and Helen E Townley**

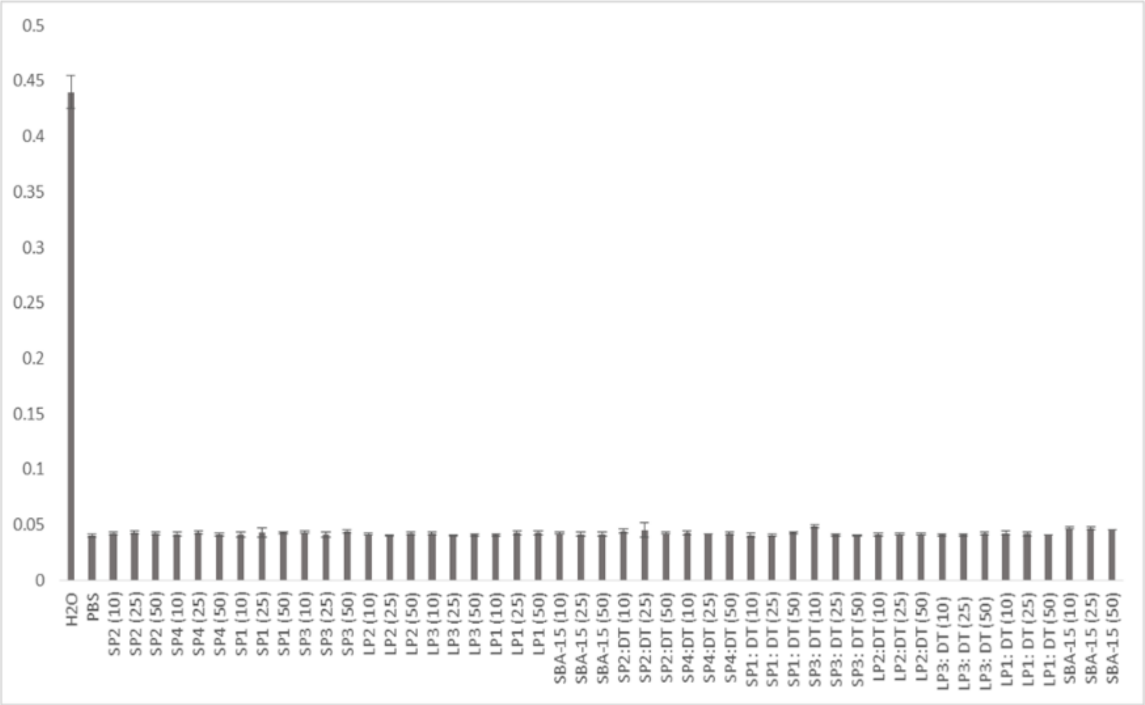


**Figure S1.** Assessment of hemolysis caused by incubation of blood with particles. Whole blood was incubated with either water, PBS, or the nanoparticles for 24 hours. The concentration of nanoparticles tested was either (10) 10 µM (25) 25 µM, or (50) 50 µM. The serum was analyzed spectroscopically to detect whether blood cell lysis had occurred. Data shown is average ± standard deviation, for triplicate samples.

Figure S2(a)

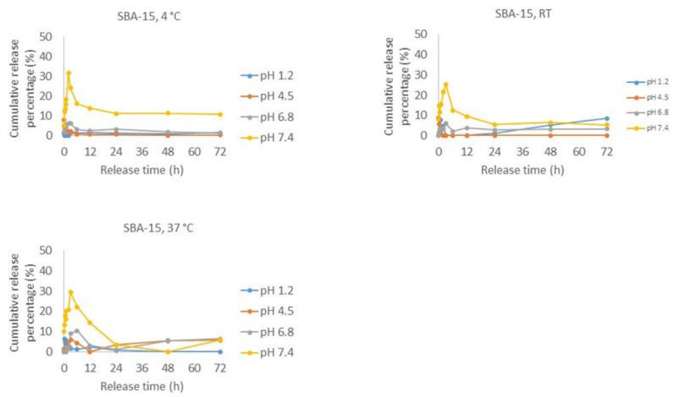


Figure S2(b)

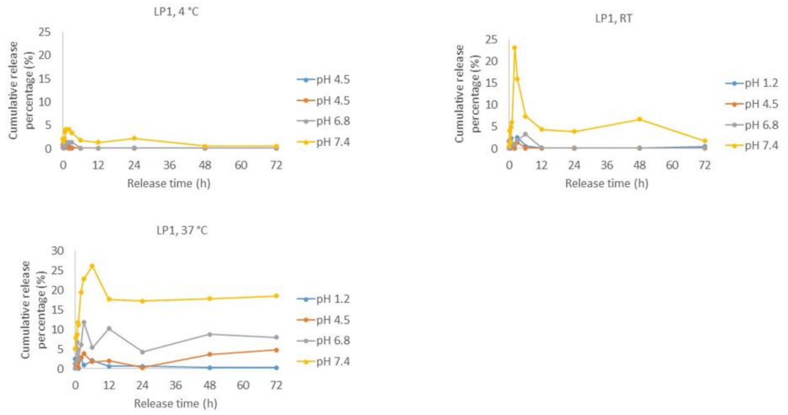


Figure S2(c)

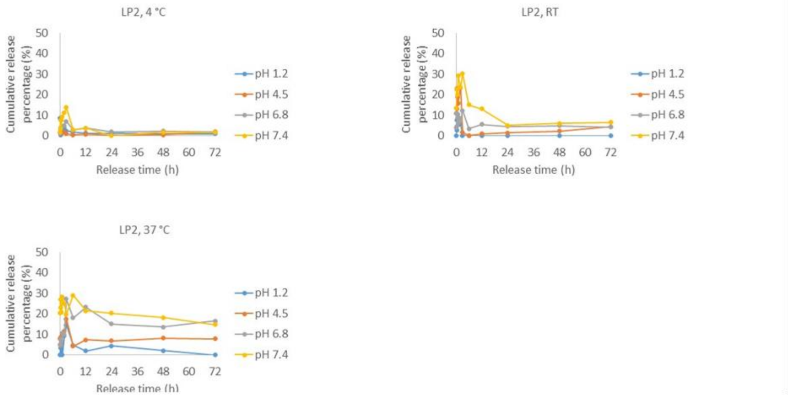


Figure S2(d)

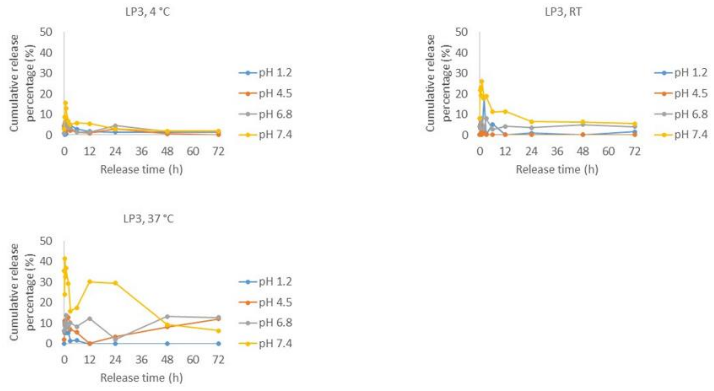


**Figure S2.** Time course to show toxoid release. Toxoid release was monitored over 72 hours at four different pH values (pH 1.2, 4.5, 6.8 and 7.4). (**a**) SBA-15 nanoparticles (**b**) LP1 (**c**) LP2 (**d**) LP3.

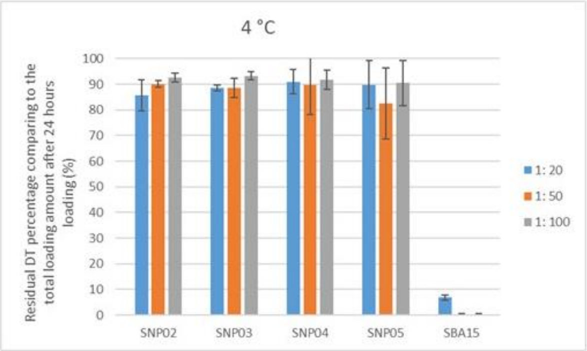


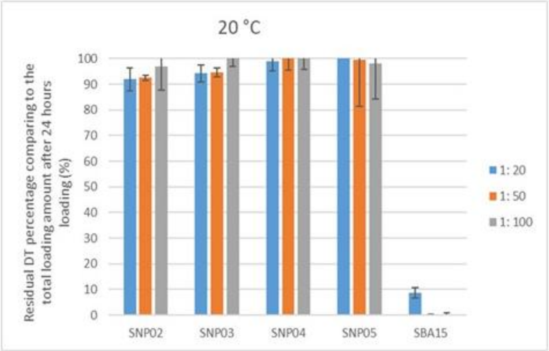


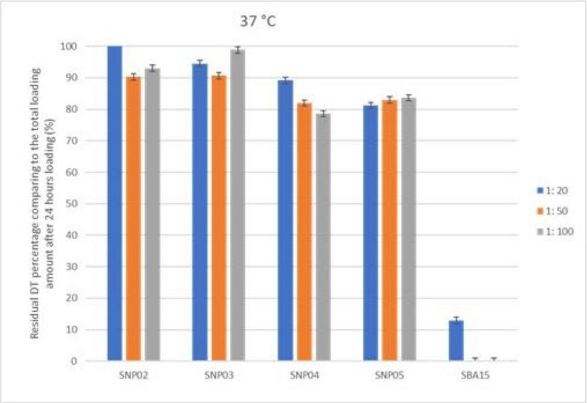


| | Size (nm) | ζ potential (mV) |
|---|---|---|
| SNP2 | 275 ± 18 | -27.3 |
| SNP3 | 378 ± 11 | -45.2 |
| SNP4 | 588 ± 26 | -45.2 |
| SNP5 | 847 ± 28 | -50.7 |

**Figure S3.** Antigen loading on solid silica nanoparticles. Diphtheria toxoid was incubated with the different MSNPs for 24 h at either 4 °C, 20 °C, or 37 °C. The toxoid to SNP ratio was varied from 1:20, 1:50 and 1:100. Graphs show the amount of unbound antigen remaining after incubation. Data shown is mean ± standard deviation. Three independent experiments were performed with triplicate samples, and data from all experiments combined.
